# Blood glucose outcomes of anti-retroviral therapy naïve Ugandan people with HIV with pre-diabetes mellitus initiated on dolutegravir for 48 weeks

**DOI:** 10.21203/rs.3.rs-3154716/v1

**Published:** 2023-08-03

**Authors:** Frank Mulindwa, Jean-Marc Schwarz, Nele Brusselaers, Martin Nabwana, Robert Bollinger, Allan Buzibye, Willington Amutuhaire, George Yendewa, Eva Laker, Ronald Kiguba, Barbara Castelnuovo

**Affiliations:** Makerere University Infectious Diseases Institute; University of California San Francisco; Antwerp University; Makerere University Johns Hopkins University Research Collaboration; Johns Hopkins University; Makerere University Infectious Diseases Institute; Case Western Reserve University; Case Western Reserve University; Makerere University Infectious Diseases Institute; Makerere University; Makerere University Infectious Diseases Institute

**Keywords:** Integrase inhibitors, Dolutegravir, Diabetes Mellitus, Blood glucose

## Abstract

**Background:**

The Uganda ministry of Health recommends frequent blood glucose monitoring for the first six months on dolutegravir, in people with HIV (PWH) having pre-diabetes mellitus (pre-DM). We sought to determine if indeed PWH with pre-diabetes started on dolutegravir had worse blood glucose outcomes at 48 weeks compared to those with normal blood glucose.

**Methods:**

In this matched cohort study, we compared 44 PWH with pre-DM and 88 PWH with normal blood glucose at baseline. The primary outcome was change in mean fasting blood glucose (FBG) from baseline to week 48 and 2-hour blood glucose (2hBG) from baseline to week 36 compared between the two groups.

**Results:**

There was significant increase in FBG in PWH with normal blood glucose (mean change in FBG(FBG): 3.9mg/dl, 95% confidence interval (95% CI): (2.2, 5.7), p value (p) = < 0.0001) and decrease in those with pre-DM (FBG: −6.1mg/dl, 95%CI (−9.1, −3.2), p = < 0.0001) at 48 weeks. 2hBG at 36 weeks was significantly lower than at baseline in both groups with the magnitude of reduction larger in those with pre-DM at 12 weeks (adjusted differences in mean drop in 2hBG (a2hBG): −19.69mg/dl, 95%CI (−30.19, −9.19), p = < 0.0001) and 36 weeks (a2hBG: −19.97mg/dl, 95%CI (−30.56, −9.39), p = < 0.0001).

**Conclusion;:**

We demonstrated that Ugandan ART naïve PWH with pre-diabetes at enrollment have consistent improvement in both fasting blood glucose and glucose tolerance over 48 weeks on dolutegravir. Intensified blood glucose monitoring of these patients in the first six months of dolutegravir may be unnecessary.

## Introduction

There has been a growing burden of non-communicable diseases (NCDs) in people with HIV (PWH) globally. Additionally, PWH have been shown to have more prevalent cardiovascular diseases compared to HIV negative persons[1], [2]. This is due to improved anti-retroviral therapy (ART) efficacy leading to improved longevity, cumulative side effects of ART, more opportunities for NCD screening for patients in HIV care and HIV infection itself[2]–[4]. As a consequence, The Joint United Nations Programme on HIV/AIDS (UNAIDS) has advocated for integrated HIV-NCD care with the aim of leveraging the existent HIV programmatic care settings to improve NCD care[5]–[7].

In 2016, the WHO recommended the use of dolutegravir (DTG), a later generation integrase strand transfer inhibitor (INSTI) as first line treatment for HIV. It was also found to be safe and effective in ART exposed PLHIV and was later recommended as an alternative second line anchor drug in 2018[8], [9]. This was due to reported wide spread primary resistance to non-nucleoside reverse transcriptase inhibitors (NNRTIs)[10]. Additionally, DTG demonstrated high efficacy, a very good side effect profile and a high genetic barrier to resistance[11]–[14]. Consequently, multiple countries in sub-Saharan Africa adopted it as first line treatment[15], [16].

Uganda adopted DTG based ART in 2018 pioneered by selected centers including the Makerere University Infectious Diseases Institute (IDI)[17], [18]. In addition to initiating DTG in ART naïve patients, those established on ART were switched to DTG based therapy as well. During the first year of use, the IDI identified sixteen cases of patients presenting with diabetic ketoacidosis (DKA) a few weeks to months post switch to DTG preceded by weight loss[19]. The majority of patients were ART exposed with only one being previously ART naïve. Subsequently, additional reports of patients taking DTG presenting with DKA have been published[20]–[23]. Due to the concern for an increased risk of asymptomatic hyperglycemia, the Uganda Ministry of Health (MoH) issued guidelines on the use of DTG. These included avoiding the use of DTG in PWH with diabetes mellitus (DM) and blood glucose monitoring every 3 months during the first six months of therapy in PWH with pre-diabetes mellitus (pre-DM) [24].

Much as most documented similar cases of accelerated severe hyperglycemia have been reported in ART exposed patients prior to the switch to dolutegravir (or other integrase inhibitors), the Uganda MoH guidelines issued were cross cutting to both ART naïve and exposed PWH[24]. We sought to determine if indeed ART naïve Ugandan PWH with pre-DM at baseline had worse blood glucose trajectories and outcomes at 48 weeks on dolutegravir compared to those with normal blood glucose at baseline and hence needed intensified blood glucose monitoring in the first months of therapy.

## METHODS

### Study design and setting.

This was a matched cohort study nested in a larger prospective cohort study, the ‘Glucose metabolism changes in Ugandan PWH on Dolutegravir’ (GLUMED study).

The GLUMED study was a prospective cohort study at the Kisenyi Health Center IV, HIV clinic in Uganda’s capital city, Kampala. The clinic is a high-volume clinic with 12,000 active PWH in care and is supported by the IDI with funding from the Center for Disease Control (CDC) and the U.S. President’s Emergency Plan for AIDS Relief (PEPFAR). Participants were recruited between 1st - January- 2021 and 20th -October – 2021 and follow up was completed in September- 2022.

### Study participants and study processes.

ART naïve PWH aged ≥ 18 years enrolling for ART care were screened for the parent (GLUMED) study inclusion. Pregnant women and patients unable to undergo a 2 hour - 75g oral glucose tolerance test (2h-OGTT) were excluded. Criteria for withdrawal during follow up included: new pregnancy and poor adherence to ART (adherence < 85% determined by pill count and self-reporting[24]). Patients with poor adherence were excluded to ascertain exposure to DTG based ART.

After providing informed consent, patients were scheduled for review in one to two days, whichever was convenient to them after an overnight fast of 8–12 hours. Baseline demographic, clinical and social data were collected which included: age, sex, CD4 count, body mass index (BMI), level of education, area of residence, blood pressure, waist circumference, tuberculosis status, smoking status, physical activity measured by the Global Physical Activity Questionnaire (GPAQ), alcohol consumption measured by the Alcohol Use Disorders Identification Test (AUDIT), serum creatine and serum lipid profiles. A 2-hour oral glucose tolerance test (2h-OGTT) was performed[25]. Patients found not to have diabetes mellitus on the 2h-OGTT (fasting blood glucose (FBG) < 126mg/dl and 2-hour blood glucose (2hBG) < 200mg/dl) were enrolled for 48-week follow up on tenofovir/ lamivudine/ dolutegravir (TDF/3TC/DTG) in line with the Uganda National HIV treatment guidelines[24]. Enrolled patients received the same adherence and positive living counselling package as the other patients in the Kisenyi HIV clinic before ART initiation.

Enrolled patients were prospectively followed up with BMI, waist circumference, adherence counselling, assessment of concurrent medications and clinical assessments at 12, 24, 36 and 48 weeks. Repeat 2h-OGTT was performed at 12 and 36 weeks while FBG was measured at 24 and 48 weeks. Viral load monitoring was performed at 24 weeks. ART adherence was evaluated on every clinical visit using self-reports and pill counts as recommended by the Uganda MoH guidelines[26]. Further details of the study are quoted in our earlier publication[27].

For this analysis we selected two cohorts at a ratio of 1:2 from the 243/ 309 participants who completed 48 week follow up in the GLUMED study. One cohort (44 PWH) comprised PWH with pre-DM defined as having either FBG of 100–125mg/dl or 2hBG of 140–199mg/dl or both at baseline. The second cohort (88 PWH) comprised PWH with normal blood glucose, defined as having both FBG of < 100mg/dl and a 2hBG of < 140mg/dl at baseline. The cohorts were matched for sex and exact or nearest age and baseline body mass index, factors known to affect glucose metabolism[28], [29]

### Outcomes

The primary outcome for this analysis was change in mean fasting blood glucose from baseline to week 48 and 2-hour blood glucose from baseline to week 36, compared between PWH with pre-DM and those with normal blood glucose at baseline.

### Statistical analysis

Details on data collection and categorization of baseline variables are given in our earlier publication[27]. Data were entered in Microsoft Excel 2016, cleaned, and transformed before it was exported for statistical analyses. Baseline characteristics were compared between participants with pre-diabetes (cases) and those with normal blood glucose (controls) using Pearson’s χ² test or Fisher’s exact test for categorical variables and median test for continuous variables. Categorical data were presented as proportions while continuous variables were presented as medians with their corresponding interquartile ranges (IQR). Mean blood glucose at each visit was compared between cases and controls using student t-test.

Multiple linear regression models were used to estimate the adjusted differences in mean blood glucose at the different time points as well as adjusted differences in mean change between baseline and post baseline blood glucose. Variables with known biological plausibility i.e. baseline age, sex, BMI, CD4 cell count and six month viral load were adjusted for in the multivariable model. Statistical significance was tested at a p-value of less than 0.05 and all p-values were two-sided. All analyses were done using Stata Release 17.0.

## RESULTS

**Figure 1:**
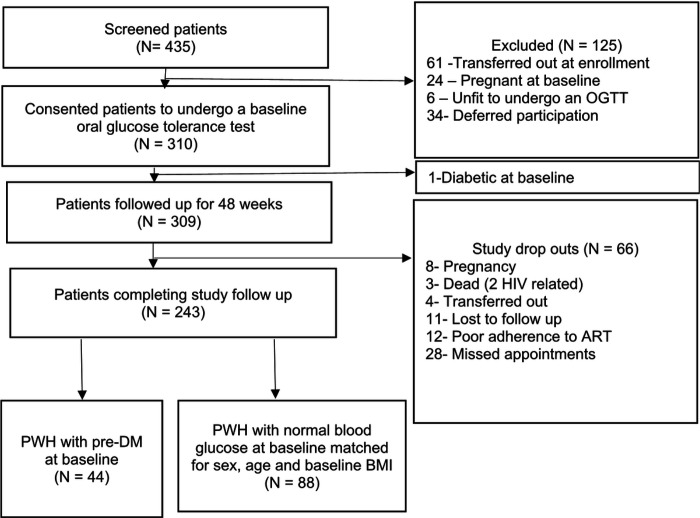
Study participant enrollment schema

Out of the 435 patients screened for enrollment, 309 patients (71.0%) were enrolled with 243 patients completing 48 weeks of follow up (21.7% drop-out) in the parent GLUMED study. Among the patients completing follow up, 44 PWH with pre-DM and 88 matched PWH with normal blood glucose at baseline were selected for this analysis (Fig. 1).

### Baseline demographic and clinical characteristics of the study participants

A total of 132 patients were evaluated. The median age of participants was 33 (Interquartile range (IQR): 27.5, 40) with 72/132 (54.5%) being females. The median baseline CD4 + cell count was 296.5 cells/mm^3^ (IQR: 160, 523.5). Sixty-seven (50.8%) of the participants had education up to primary level and 56/132 (42.4%) had studied up to secondary level. Of the evaluated participants, 122/132 (92.4%) resided in an urban setting while 10/132 (7.6%) resided in rural areas. Majority (111/132 (84.1%) had no tuberculosis (TB) symptoms, 19 (14.4%) had TB symptoms but were found not to have TB on full clinical evaluation and 2/132 (1.5%) were found to have TB and started on anti-TB therapy. One hundred twenty-seven (96.2%) were in WHO clinical stage 1, 2/132 (1.5%) in clinical stage 2, 2/132 (1.5%) in clinical stage 3 and 1/132 in clinical stage 4. On baseline BMI assessment, 81/132 (61.4%) had a normal BMI, 34/132 (25.8%) were overweight, 5/132 (3.8%) were obese and 12/132 (9.1) were underweight. Most (103/132 (78%)) of the patients met the WHO requirements for physical exercise. At 24 weeks, 130/132 (99.2%) were virologically suppressed. Median baseline serum creatine was 0.84mg/dl (IQR0.76, 0.97).

There were no significant differences between the two patient cohorts for all baseline parameters mentioned above (Table 1)

### Fasting blood glucose trajectories and mean differences at different time points compared between PWH with normal blood glucose and those with pre-DM at baseline, adjusted for sex, age, CD4 + cell count, 24-week viral load and BMI.

**Figure 2:**
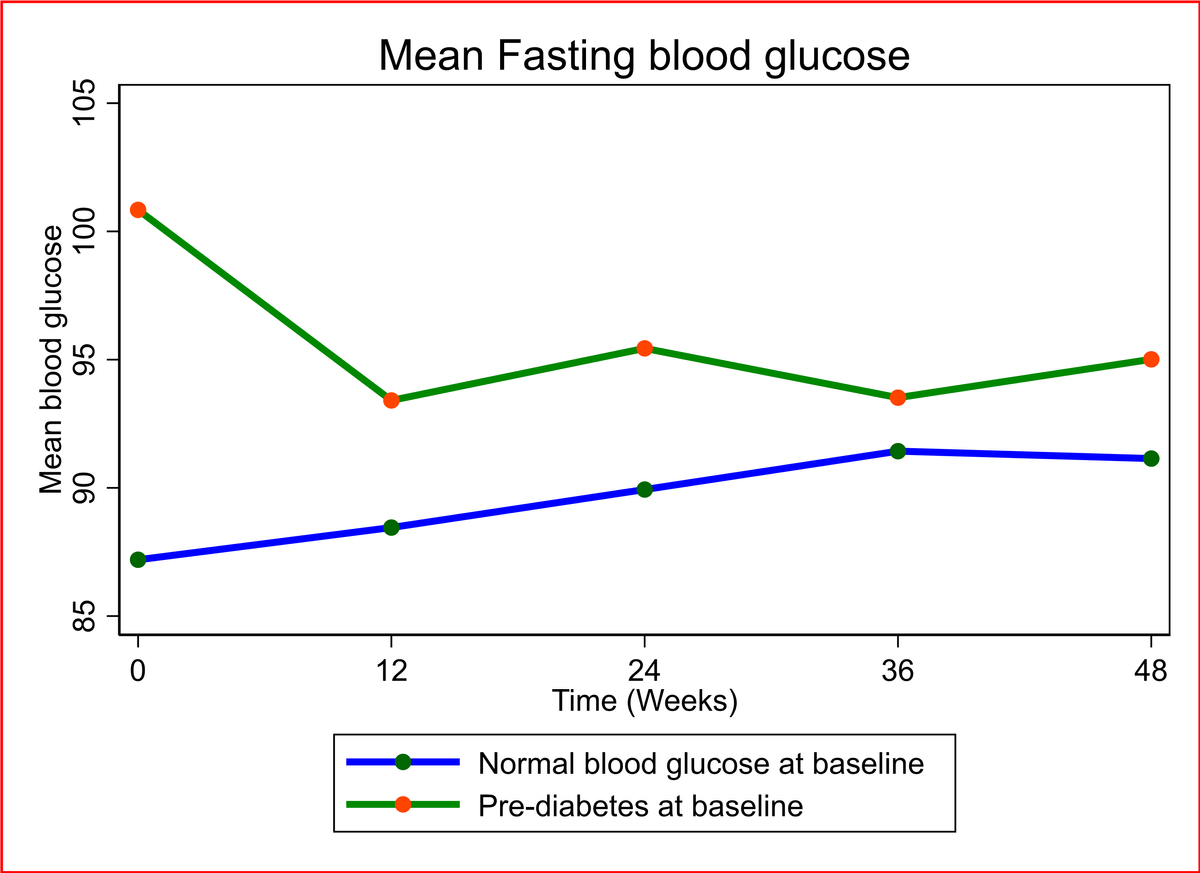
Mean Fasting blood glucose over 48 weeks compared between participants with pre-diabetes mellitus and those with normal blood glucose at baseline. Blood glucose= mg/dl

There was a significant increase in fasting blood glucose in PWH with normal blood glucose from baseline to week 48 (adjusted mean change in FBG(aFBG): 3.9 mg/dl, 95% confidence interval (95% CI): (2.2, 5.7), p value (p) = < 0.0001) and a significant reduction in fasting blood glucose in those with pre-DM from baseline to week 48 weeks (aFBG: −6.1mg/dl, 95%CI (−9.1, −3.2), p = < 0.0001) (Table S1-supplementary material, Fig. 2). Despite the reduction in fasting blood glucose, FBG was higher in the participants with pre-DM than in those with normal baseline blood glucose at 12 weeks (adjusted difference in FBG between the two groups (aFBG); 4.9 mg/dl, 95%CI (1.5, 8.3), p = 0.005), 24 weeks (aFBG: 5.4 mg/dl, 95%CI (2.5, 8.3), p = < 0.0001) and 48 weeks (aFBG: 4.0 mg/dl, 95%CI (1.3, 6.7), p = 0.004) apart from at 36 weeks (aFBG:2.2 mg/dl, 95%CI (−0.2, 4.7), p = 0.075) (Table 2).

### 2-hour blood glucose trajectories and mean differences at different time points compared between PWH with normal blood glucose and those with pre-DM at baseline, adjusted for sex, age, CD4 + cell count, 24-week viral load and BMI.

**Figure 3:**
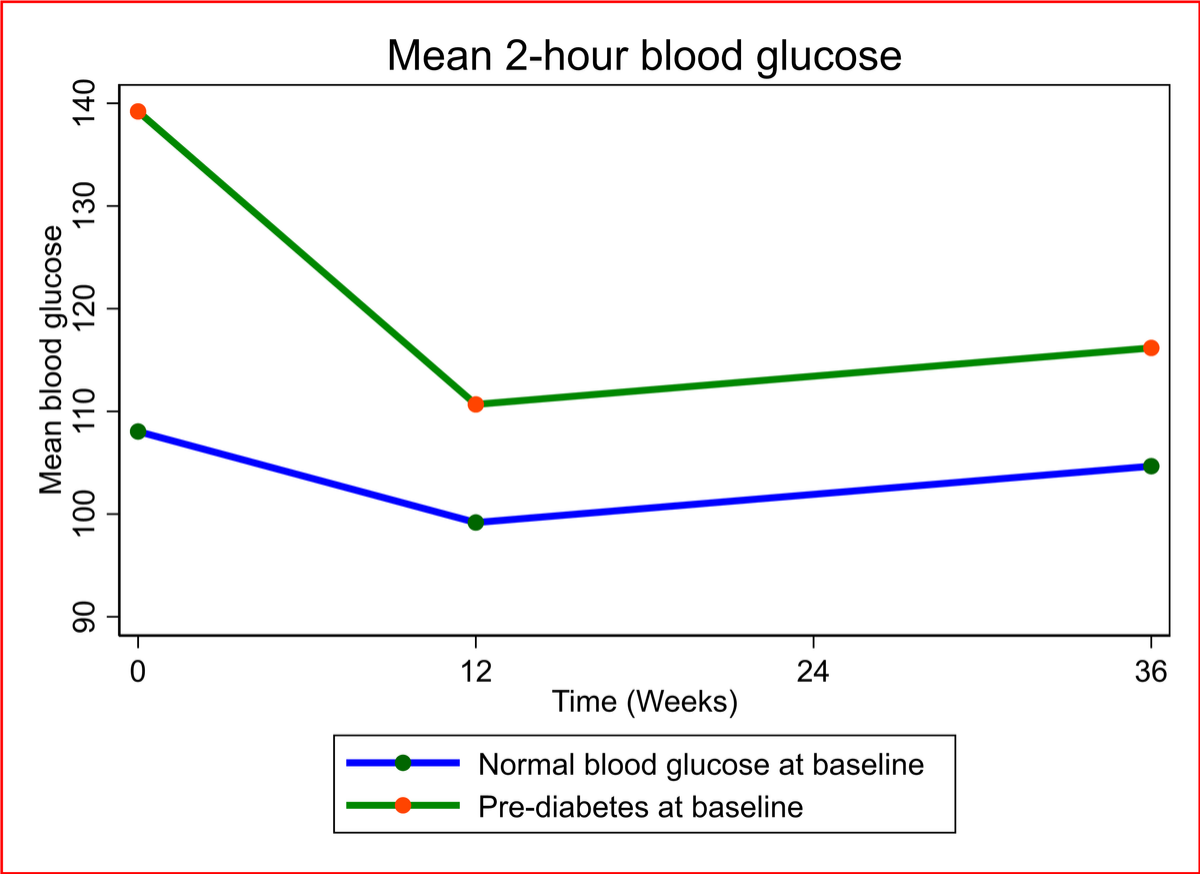
Mean 2-hour blood glucose over 36 weeks compared between PWH with pre-diabetes mellitus and those with normal blood glucose at baseline. Blood glucose= mg/dl

2hBG at week 36 was significantly lower than at baseline in both participants with pre-DM at baseline (adjusted mean change in 2hBG at 36 weeks from baseline (a2hBG: −23.0 mg/dl, 95%CI (−33.4, −12.6), p = 0.0001) and those with normal blood glucose at baseline (a2hBG: −3.4 mg/dl, 95%CI (−8.6, 1.8), p = 0.1988). (Table S1-supplementary material). Despite the drop in 2hBG in both groups, 2hBG was significantly higher in those with pre-DM versus those with normal blood glucose at 12 (adjusted difference in mean 2hBG between both groups (a2hBG): 10.9 mg/dl, 95%CI (3.5, 18.4), p = 0.005) and 36 weeks (a2hBG: 10.7 mg/dl, 95%CI (2.6, 18.7), p = 0.01) (Table 2, Fig. 3).

The magnitude of the drop in 2hBG was significantly greater in PWH with pre-DM at baseline at 12 weeks (adjusted differences in mean drop in 2hBG (a2hBG): −19.69 mg/dl, 95%CI (−30.19, −9.19), p = < 0.0001) and 36 weeks (a2hBG: −19.97 mg/dl, 95%CI (−30.56, −9.39), p = < 0.0001). (Table 3, figure S1-supplementary material).

## DISCUSSION

We carried out a matched cohort study to determine if ART naïve Ugandan PWH who had pre-diabetes mellitus at enrollment when exposed to dolutegravir for 48 weeks had worsening blood glucose hence requiring more frequent blood glucose monitoring than patients with normal blood glucose. We demonstrated that Ugandan PWH with pre-diabetes had consistent improvement in both FBG and 2hBG over 48 weeks and the magnitude of improvement was significantly higher compared to those with normal blood glucose at baseline. We also demonstrated that FBG at 48 weeks and 2hBG at 36 weeks was significantly lower than at baseline.

The current Uganda Ministry of Health HIV treatment guidelines stratify ART naïve and experienced PWH with pre-DM and risk factors for diabetes at baseline as being at heightened risk of incident DM in the first year of DTG therapy and hence recommend three monthly blood glucose monitoring for the first six months of treatment[24]. Contrary to what is believed to be the highest-risk period, in our study we demonstrated that the biggest magnitude of improvement in both FBG and 2hBG was in the first 3–6 months of therapy.

Heightened systemic inflammation typical of untreated HIV is known to disrupt insulin signaling at end organs including skeletal muscle, adipocytes and the liver leading to insulin resistance as well as impaired insulin production[30]–[32]. There is evidence documenting reduction in systemic inflammation when ART is introduced in PLHIV[33], [34]. Some studies have suggested the reduced inflammation is even more evident in patients on integrase inhibitors compared to other ART classes[35],[30]. Introduction of ART with reduced systemic inflammation may explain the dramatic improvement in glucose tolerance in the first 12 weeks in both groups. Despite of the improving blood glucose trajectories and 48-week outcomes, consistently the PWH with pre-DM at baseline had significantly higher blood glucose through 12,24,36 and 48 weeks. This could be explained by the fact that inherently this group may have had more pronounced insulin resistance and or pancreatic beta cell dysfunction that remained consistently higher even with the introduction of ART.

Studies in North America and Europe comparing fasting blood glucose trajectories in PWH on INSTIs, NNRTIs and PIs have generally demonstrated modest but significant blood glucose improvement in the first 24 to 48 weeks in patients on INSTI but insignificantly different from other drug classes[36]–[38]. The magnitude of improvement in FBG was more pronounced in the participants with pre-DM in our study compared to the quoted studies but it is important to note the difference in population demographics as well as study settings.

In an earlier meta-analysis evaluating the association of INSTI use with incident diabetes mellitus, we demonstrated reduced risk in comparison to NNRTIs and protease inhibitors (PIs) apart from African populations where the risk was threefold increased[39]. These results have been replicated in another published meta-analysis[40]. One shortcoming of our analysis was the under-representation of African populations in the eligible studies. Following that analysis, we demonstrated that in the first year of dolutegravir use in Ugandan ART- naïve PWH, the incidence of diabetes mellitus was very low, comparable if not less than what is reported in safety data of landmark DTG efficacy trials and population-based cohorts[27]. Additionally, patients had improving glucose tolerance throughout the 48 weeks of follow up. The current analysis suggests Ugandan ART naïve patients with pre-diabetes at baseline, as well have reassuring blood glucose trajectories and outcomes over 48 weeks of DTG therapy.

We had various limitations in our study: Very sick patients were not enrolled in the parent study (GLUMED study) which patients may have different glucose metabolism dynamics due to heightened inflammation. Given the participants were aware they were being monitored for blood glucose changes, they may have positively modified their lifestyles giving better blood glucose trajectories than they would have appeared in real life non-research settings. Despite that, we had a number of strengths. Both patient groups that were compared were well matched for factors that can confound glucose metabolism such as sex, BMI, alcohol consumption, physical exercise, renal function, tuberculosis status, CD4 + cell count as well as HIV virologic suppression at 24 weeks. We had a clearly defined metabolic outcome with the use of a 2-hour OGTT, a sensitive test for evaluation of glucose tolerance.

## CONCLUSION

We demonstrated that Ugandan ART naïve PWH with pre-diabetes mellitus at enrollment onto dolutegravir have consistent improvement in both fasting blood glucose and glucose tolerance over 48 weeks and this improvement is most marked in the first 12 weeks. Intensified blood glucose monitoring of these patients in the first six months of DTG anchored ART may be unnecessary.

## Figures and Tables

**Table 1:** Baseline clinical and demographic characteristics compared between participants with pre-diabetes mellitus and those with normal blood glucose at baseline.

Characteristic	Total N = 132	PWH with pre-DM at baseline n = 44	PWH with normal blood glucose at baseline n = 88	P-value
**Age** (Years), *Median (IQR)*	33 (27.5, 40)	33.5 (27.5, 40)	33 (27.5, 40)	0.902
**Sex N (%)**			
Female	72 (54.5)	24 (54.5)	48 (54.5)	
Male	60 (45.5)	20 (45.5)	40 (45.5)	0.573
**Baseline CD4 cell count** (cells/mm^3^), *Median (IQR)*	296.5 (160, 523.5)	281 (208, 507)	299 (160, 524)	0.836
**Level of education N (%)**				
Uneducated	1 (0.8)	0 (0)	1 (1.1)	
Primary	67 (50.8)	22 (50)	45 (51.1)	
Secondary	56 (42.4)	19 (43.2)	37 (42)	
Tertiary	8 (6.1)	3 (6.8)	5 (5.7)	0.901
**Religion N (%)**				
Christian	104 (78.8)	31 (70.5)	73 (83)	
Muslim	28 (21.2)	13 (29.5)	15 (17)	0.098
**Residence N (%)**				
Rural	10 (7.6)	3 (6.8)	7 (8)	
Urban	122 (92.4)	41 (93.2)	81 (92)	0.816
**Employment N (%)**				
No	17 (12.9)	3 (6.8)	14 (15.9)	
Yes	115 (87.1)	41 (93.2)	74 (84.1)	0.142
**Marital status N (%)**				
Single	72 (54.5)	23 (52.3)	49 (55.7)	
Married	60 (45.5)	21 (47.7)	39 (44.3)	0.711
**Tuberculosis status N (%)**				
No symptoms	111 (84.1)	38 (86.4)	73 (83)	
TB suspect	19 (14.4)	6 (13.6)	13 (14.8)	
TB disease	2 (1.5)	0 (0)	2 (2.3)	0.587
**Baseline blood pressure**				
Normal BP	91 (68.9)	29 (65.9)	62 (70.5)	
Pre-hypertension	26 (19.7)	9 (20.5)	17 (19.3)	
Hypertension	15 (11.4)	6 (13.6)	9 (10.2)	0.816
**HIV clinical stage**				
Stage 1	127 (96.2)	42 (95.5)	85 (96.6)	
Stage 2	2 (1.5)	1 (2.3)	1 (1.1)	
Stage 3	2 (1.5)	0 (0)	2 (2.3)	
Stage 4	1 (0.8)	1 (2.3)	0 (0)	0.474
**Body Mass Index (BMI) kg/m^2^, N (%)**				
Underweight (< 18.5)	12 (9.1)	3 (6.8)	9 (10.2)	
Normal (18.5–24.9)	81 (61.4)	25 (56.8)	56 (63.6)	
Overweight (25.0–29.9)	34 (25.8)	13 (29.5)	21 (23.9)	
Obese (≥ 30)	5 (3.8)	3 (6.8)	2 (2.3)	0.484
**Waist circumference (cm), N (%)**				
Normal	89 (67.4)	27 (61.4)	62 (70.5)	
Increased risk of cardiometabolic complications	24 (18.2)	6 (13.6)	18 (20.5)	
Substantially increased risk of cardiometabolic complications	19 (14.4)	11 (25)	8 (9.1)	0.044
**Smoking status N (%)**													
Smoker	5 (3.8)	2 (4.5)	3 (3.4)	
Non-smoker	127 (96.2)	42 (95.5)	85 (96.6)	0.542
**Physical activity N (%)**				
GPAQ < 600 MET minutes	29 (22)	13 (29.5)	16 (18.2)	
GPAQ ≥ 600 MET minutes	103 (78)	31 (70.5)	72 (81.8)	0.137
**Alcohol consumption N (%)**				
No consumption	81 (61.4)	26 (59.1)	55 (62.5)	
Low risk alcohol consumption	34 (25.8)	11 (25)	23 (26.1)	
Hazardous alcohol consumption	10 (7.6)	3 (6.8)	7 (8)	
Risk of alcohol dependence	7 (5.3)	4 (9.1)	3 (3.4)	0.611
**24-week viral load, copies/ml N (%)**				
Virologically suppressed	130 (99.2)	43 (100)	87 (98.9)	
Unsuppressed Viral load	1 (0.8)	0 (0)	1 (1.1)	0.672
**Laboratory investigations, Median (IQR)**				
Creatinine (mg/dl)	0.84 (0.76, 0.97)	0.87 (0.76, 1.04)	0.84 (0.76, 0.94)	0.409
Low Density Lipoproteins (mg/dl)	77.3 (59.9, 89.3)	81.8 (65.4, 94.0)	75.4 (58.0, 88.2)	0.113
High Density Lipoproteins (mg/dl)	31.7 (25.5, 40.2)	30.9 (25.1, 38.3)	32.9 (27.1,40.2)	0.487
Total cholesterol (mg/dl)	133.8 (116.0, 159.3)	140.0 (120.3, 158.2)	133.0 (114.5, 159.3)	0.339
Triglycerides (mg/dl)	89.5 (66.4, 117.8)	81.9 (62.0,133.8)	90.4 (69.1, 116.9)	0.571

PWH-People with HIV, MET-*Metabolic Equivalent of Task, IQR-Interquartile range, GPAQ-Global Physical Activity questionnaire, N = Total number of participants, CD4-Cluster of Differentiation 4.*

**Table 2:** Mean blood glucose at different time points compared between participants with pre-diabetes mellitus and those with normal blood glucose at baseline

Characteristic	Pre-diabetes	Normal blood glucose	Difference	P-value	*Adjusted difference	P-value
	Mean (95% CI)	Mean (95% CI)	Mean (95% CI)			
**Fasting blood glucose**					
Baseline	100.8 (97.9, 103.8)	87.2 (85.8, 88.6)	13.6 (10.8, 16.5)	< 0.0001	13.6 (10.8, 16.4)	< 0.0001
Week 12	93.4 (90.5, 96.3)	88.5 (86.6, 90.3)	5.0 (1.7, 8.3)	0.0035	4.9 (1.5, 8.3)	0.0050
Week 24	95.4 (92.4, 98.5)	89.9 (88.6, 91.3)	5.5 (2.7, 8.4)	0.0002	5.4 (2.5, 8.3)	< 0.0001
Week 36	93.5 (91.3, 95.7)	91.4 (90.0, 92.8)	2.1 (−0.4, 4.6)	0.0980	2.2 (−0.2, 4.7)	0.0750
Week 48	95.0 (92.5, 97.5)	91.1 (89.7, 92.6)	3.9 (1.2, 6.5)	0.0048	4.0 (1.3, 6.7)	0.0040
**2-hour OGTT glucose**					
Baseline	139.2 (130.7, 147.7)	108.0 (103.5, 112.6)	31.2 (22.5, 39.8)	< 0.0001	30.6 (21.9, 39.4)	< 0.0001
Week 12	110.7 (104.6, 116.7)	99.2 (95.0, 103.3)	11.50 (4.3, 18.7)	0.0020	10.9 (3.5, 18.4)	0.0050
Week 36	116.2 (107.4, 125.0)	104.7 (100.9, 108.4)	11.5 (3.5, 19.6)	0.0053	10.7 (2.6, 18.7)	0.0100

Pre-DM-pre-diabetes mellitus, CI-confidence interval, OGTT-Oral glucose tolerance test.

* Adjusted for baseline age, BMI, CD4 + cell count, sex and 24-week viral load.

**Table 3:** Differences in mean Changes in fasting and 2-hour blood glucose between participants with normal blood glucose and those with pre-diabetes mellitus over 48 weeks

Characteristic	Pre-diabetes	Normal blood glucose	Difference	P-value	*Adjusted difference in mean changes	P-value
	Mean change (95% CI)	Mean change (95% CI)	Mean change (95% CI)			
**Fasting blood glucose**					
Baseline to week 12	−7.43 (−10.33, −4.54)	1.25 (−0.74, 3.25)	−8.69 (−12.14, −5.24)	< 0.0001	−8.69 (−12.24, −5.15)	< 0.0001
Baseline to week 24	−5.40 (−8.88, −1.92)	2.74 (1.13, 4.35)	−8.14 (−11.45, −4.83)	< 0.0001	−8.20 (−11.61, −4.79)	< 0.0001
Baseline to week 36	−7.32 (−9.90, −4.75)	4.23 (2.62, 5.85)	−11.56 (−14.45, −8.67)	< 0.0001	−11.36 (−14.27, −8.44)	< 0.0001
Baseline to week 48	−6.12 (−9.08, −3.16)	3.95 (2.16, 5.73)	−10.07 (−13.32, −6.82)	< 0.0001	−10.03 (−13.25, −6.82)	< 0.0001
**2-hour OGTT glucose**					
Baseline to week 12	−28.53 (−38.97, −18.10)	−8.86 (−14.12, −3.61)	−19.67 (−30.01, −9.33)	0.0003	−19.69 (−30.19, −9.19)	< 0.0001
Baseline to week 36	−23.03 (−33.44, −12.63)	−3.38 (−8.56, 1.81)	−19.65 (−29.91 (−9.30)	0.0002	−19.97 (−30.56, −9.39)	< 0.0001

CI-confidence interval, OGTT-Oral glucose tolerance test.

* Adjusted for baseline age, BMI, CD4 + cell count, sex and 24-week viral load.

## LIST OF ABBREVIATIONS

HIV: Human Immunodeficiency Virus
NCD: Non-communicable Diseases
UNAIDS: The Joint United Nations Programme on HIV/AIDS
INSTI: Integrase Strand Transfer Inhibitors
MoH: Ministry of Health
PEPFAR: U.S. President’s Emergency Plan for AIDS Relief
ART: Anti-retroviral Therapy
NNRTI: Non- Nucleoside Reverse Transcriptase Inhibitors
DTG: Dolutegravir
WHO: World Health Organization
IDI: Infectious Diseases Institute
DKA: Diabetic Ketoacidosis
T2DM: Type 2 Diabetes Mellitus
Pre-DM: Pre-diabetes Mellitus
PWH: People with HIV
OGTT: Oral Glucose Tolerance Test
CDC: Center for Diseases Control
BMI: Body Mass Index
GPAQ: Global Physical Activity Questionnaire
AUDIT: Alcohol Use Disorders Identification Test
FBG: Fasting Blood Glucose
2hBG: 2-hour Blood Glucose
MET: Metabolic Equivalent of Task
